# Authors' Responses to Peer Review of “Machine Learning and Medication Adherence: Scoping Review”

**DOI:** 10.2196/33962

**Published:** 2021-11-24

**Authors:** Aaron Bohlmann, Javed Mostafa, Manish Kumar

**Affiliations:** 1 Carolina Population Center University of North Carolina at Chapel Hill Chapel Hill, NC United States; 2 Public Health Leadership Program University of North Carolina at Chapel Hill Chapel Hill, NC United States

*This is the authors’ response to peer-review reports for the paper “Machine Learning and Medication Adherence: Scoping Review”.*

## Round 1 Review

### Reviewer F [1]

#### General Comments

This paper [2] covers a very interesting area on the use of machine learning for assessment of medication adherence, yet in its current version, it does not add a lot to the field. It is a pity, as it seems that the authors performed their review well. However, the presentation of the results is not acceptable.

#### Major Comments

1. It creates a lot of confusion that the authors use “adherence” instead of “compliance.” In fact, these two are equivalent terms, of which adherence is preferred and compliance is a bit old-fashioned. The authors need to define the major concept they use, and these two need to be carefully checked against available literature and the ABC taxonomy.
  *Response:* Definition explained under the updated Methods Eligibility Criteria section.
  This taxonomy defines medication adherence as “The process by which patients take their medications as prescribed, composed of initiation, implementation and discontinuation” [3].
2. The Abstract provides no numeric data; even the number of identified publications is missing. Similarly, the conclusions of the Abstract are inconclusive.
  *Response:* This point is addressed mostly in the updated Abstract and the updated Discussion/Conclusions section.
3. The authors mentioned previous reviews in this area, yet they did not make it clear what was different about their own work. What exactly was missing in the previous reviews that turned them toward this new exercise?
  *Response:* This is addressed under Introduction, paragraphs three and four.
4. Publication selection for review: What were the criteria used to identify acceptable papers in the full-text review? What was the reason for screening a sample of 20 papers first?
  *Response:* Updated the eligibility criteria and selection of sources of evidence sections to address this issue.
5. “Medication adherence activities” is not a term used in the literature to describe interventions aimed at assessment or modification of medication adherence. Please use another term that is used in the existing literature.
  *Response:* I have determined that the creation of a new term is not necessary to explain my ideas in this part of the paper. I have changed medication adherence activities to verbs related to medication adherence. In this way, I can explain my idea without introducing new terminology that is potentially confusing for the reader. The changes are located in the analysis of natural categories paragraph and throughout the manuscript.
6. The paper is lacking a lot of details; for example, what was the basis for the dichotomization of the source databases into “biomedical” and “computer” in Figure 3?
  *Response:* This part of the analysis has been removed to reduce confusion and to allow more room for a figure depicting the article review process (Figure 1).
7. In Tables 1-3, instead of simply providing the number of the reference, it is also advisable to have the first author’s name and the year of the publication.
  *Response:* I added the date of publication for all three tables. I have decided against adding the author’s name because I do not know what it would add to the publication, and it was not a part of the original data charting document. Additionally, the tables barely fit neatly on the page as is, making it very difficult to add any additional information.
8. I have a feeling that the studies listed in Table 1, based on self-report and pharmacy claims data, do not “predict” adherence but rather assess it. Please correct me if I am wrong.
  *Response:* All of the studies listed in Table 1 use machine learning to actually predict medication adherence in the future. These predictions are made according to past patient information (age, sex, socioeconomic status, etc) and their level of medication adherence. Claims data from the past can be used in combination with personal information to build models that predict if a specific patient will be adherent to their medication in the future.
9. The paper must be self-explanatory; therefore, abbreviations such as DOT need to be explained. When addressing a general audience, it makes sense to do the same with the abbreviations of algorithms cited within.
  *Response:* The paper has been reviewed to either eliminate or explain all abbreviations. However, some algorithms look like abbreviations but that is simply the name of the algorithm, so this may still be somewhat confusing. For example, J48 is the full name of an algorithm and is not an abbreviation.
10. Numbers, numbers, numbers, please! The Results section reads, for example, “LEAP had the best prediction accuracy of the machine learning methods used”—by how much? Was the difference statistically significant?
  *Response:* I went through the paper and greatly increased the number of numbers and citations. This is particularly evident in the updated Abstract and the updated Discussion/Conclusions section. When different algorithms were used, I stated the prediction accuracy, as this was the most frequently reported metric. Most of the papers that I state the main results of did not specifically say if results were statistically significant or not so this was not added to the paper.
11. Being a clinician, I feel that this might be information technology (IT) jargon: “The first of these articles used data collected during hospital stays to generate features” (from Results). However, please make sure that the text is also meaningful for non-IT people.
  *Response:* Feature and features are IT jargon. Features are variables selected from a data set for further analysis using machine learning techniques. I have changed all mentions of feature or features to predictor or predictors since this is a synonym that provides more insight for non-IT people.
12. In light of previous publications in the field, the first sentence of the Discussion needs to be rechecked.
  *Response:* To my knowledge, this is the first scoping review to focus broadly on using machine learning for medication adherence activities. There are other reviews in this field, but they are typically much more narrowly focused, probably because they are systematic reviews and not scoping reviews.
13. In the Discussion, the authors say “However, more work needs to be done to better understand the impact of socioeconomic status [on adherence].” In fact, a lot of work has been done in that area, and it would help the paper if authors would broaden their understanding of it.
  *Response:* I am not trying to say that there has not been a lot of work examining the impact of socioeconomic status on adherence. I am explaining that machine learning algorithms are not currently leveraging this information enough. This point has been clarified in the Discussion/Conclusions section, paragraph three.
14. From the Discussion: “Some of these works compared the different types of algorithms to determine which was the most accurate...” Which ones? Please cite!
  *Response:* I see why this is confusing after reading your comment. The studies comparing algorithms are listed in the tables with a reference number, but it does not specify if the multiple algorithms listed indicate a comparison or if different algorithms in combination make a single prediction. This point has been clarified in the Discussion/Conclusions section.
15. To conclude, it needs to be stressed that the authors should extract a lot more data and conclusions from the material they reviewed—instead of saying “some studies...,” please provide the numbers (eg, “over 40% of studies found the parameter to change by >90%”).
  *Response:* This point has been addressed in the updated Abstract and the updated Discussion/Conclusions section.

### Reviewer BQ [4]

#### General Comments

This paper aims to categorize and summarize literature focused on using machine learning for medication compliance activities. There are major concerns associated with this paper.

#### Major Comments

1. The aim does not feel like an actual aim. I would suggest saying things like “aim to do a scoping review on... and categorize and summarize...”
  *Response:* The Introduction paragraphs three and four have been updated to address this point.
2. You should state the design in the Methods. In addition, you should state clearly the inclusion and exclusion criteria. As of now, the inclusion and exclusion criteria are too broad to do a robust review.
  *Response:* This is a scoping review, so the inclusion criteria are supposed to be more broad than a systematic review. I have added more detail in the eligibility criteria and selection of sources of evidence sections to address this issue.
3. Is it adherence or compliance? The frequent change of terms makes it hard to understand what the authors want to do. They are very different fundamentally.
  *Response:* Medication adherence is used throughout the manuscript to reduce confusion.
4. Limitations should be before the Conclusion.
  *Response:* The Limitations section was moved before the Discussion/Conclusions section.
5. They lack Figure 1: the number of articles screened/reviewed.
  *Response:*Figure 1 in the appendix was moved into the paper and explains the screening/review process in detail.
6. Figure 2 is not right; there are many overlapping diseases in each category.
  *Response:*Figure 2 was updated by grouping diseases instead of listing them separately.
7. Short forms are not well explained or mentioned in the tables.
  *Response:* Explanations have been added to explain short forms in the table. The explanations are mentioned throughout the manuscript when the abbreviated term first appears.

## Round 2 Review

### Reviewer F

#### Major Comments

1. In the body of text, you refer to the help and advice provided by two librarians and two pharmacists, yet it seems to me that they are not included in the authorship nor thanked in the Acknowledgments. Please take care of solving this.
  *Response:* In the Search Query section, I have added the names of the librarians that helped with query creation.
2. Overall, the interesting work done in this exercise is not followed with a clear description. In fact, it is very hard to learn what exactly the use of machine learning was in the context of medication adherence or the outcomes of this process. These, however, were the major objectives of this paper. In such a case, the conclusion from the Abstract stating that “Machine learning has the potential to greatly improve medication adherence” seems to be unsupported by the data presented.
  *Response:* I agree with this comment and have extensively reworked the Discussion and Conclusion sections to address this issue. Additionally, a column has been added to Tables 1-3 to provide the main outcome metric for each study. This addition of the outcome metric and further explanation of how the systems work have allowed me to provide more context on the current status of these systems and some insight into their future potential. I also extensively reworked the Abstract to address this point.

#### Additional Suggestions

- Line 23: The number of identified studies belongs in the Results.
  *Response:* The explanation of how studies were selected is located in the Selection of Sources of Evidence section per the recommendation of the PRISMA (Preferred Reporting Items for Systematic Reviews and Meta-Analyses) scoping review guidelines. I added a brief mention of the number of selected studies in the Results of Individual Sources of Evidence section as well.
- Line 26 onward: “Verb” is an uncommonly used term in this context; please search the literature to find a more frequently used equivalent.
  *Response:* After talking this point over with the coauthors (MK and JM), we have decided to change the terms to either action or actions. We have determined that these terms are the most easily understood within this context.
- Line 29 onward: Using percentiles makes sense when the total number is ≥100; in this case, the number of identified publications was only 43; what justifies fractions and not the percentiles?
  *Response:* Percentages were changed to fractions or individual numbers where appropriate throughout the Results sections.
- Lines 42-3: The Discussion is missing in the Abstract (what is provided now is not a real discussion of the findings).
  *Response:* The Discussion has been extensively reworked to address this issue.
- Lines 92-3: The eligibility criteria need to be more detailed; it is unclear now what sort of relationship had to link the included publications with medication adherence, and what was the exclusion criteria?
  *Response:* More detail has been added to the following sections to address this comment: Eligibility Criteria, Search Query, and Selection of Sources of Evidence.
- Line 134 refers to “predictors”—predictors of what?
  *Response:* These are predictors of medication adherence. Medication adherence has been added here to clear up this confusion.
- Line 136-7: What do you mean by “The data collected for this study was qualitative and sometimes quantitative”? What does “sometimes” mean in this context?
  *Response:* After discussion with one of the coauthors (MK), this sentence has been removed. He concluded correctly that I was simply talking about numerical and text data, which does not need to be mentioned specifically.
- Line 165 refers to “13 studies,” yet Figure 3 shows only 12 items in that category.
  *Response:* I have corrected the number from 13 to 12 in the text.
- Tables 1-3 need serious improvement. Putting all the comments together in columns placed to the right makes no sense. No idea why “Some entries were excluded for brevity,” especially in cases of short algorithm acronyms. The footnote marked ** is not applicable to Table 2.
  *Response:* For Table 1, the merged cells were removed to provide more specific detail from each study. The summarized findings are still presented later in the writing, so it does not need to be expressed twice. All algorithms were included in Table 1 to address this comment, and the ** footnote was removed from Tables 1 and 2.
- Table 1: I would love to see one more column describing what sort of adherence measure the machine learning algorithm was able to predict (eg, “filling the prescription” or “daily drug intake”).
  *Response:* Removed the limitations column and replaced it with the adherence metric predicted column. The information in the Limitations section is stated later in writing, so it seemed redundant. I also agree that the adherence metric predict column provides readers with more insight about the individual studies in Table 1.
- Table 1: How did you identify the “strong predictors”? Has any statistical threshold been applied to this selection?
  *Response:* Strong predictors were identified in the individual studies, and this process varies depending on the methods used in each study. Typically, the strongest predictors are chosen by adding and or removing variables from the prediction algorithm until the highest prediction accuracy was achieved. However, this has to be balanced against overfitting, which is when the algorithm is trained too closely to the test data and performs very poorly when tested using a new data set. Often algorithms become overfit when too many predictor variables are included. Certain algorithms can also provide a strength metric for every variable allowing easy comparison of the strength of each predictor. Some studies did mention the statistical significance of specific predictors, but this was not common.
  I have added a footnote to Table 1 that states that predictor strength was based on individual study results to clarify that this study did not determine the strength of different predictors.
- Table 2: I would love to see one more column describing what sort of adherence measure the machine learning algorithm was able to identify. For example, there are plenty of studies using smart pill bottles—so what exactly was the role of machine learning in [5] for it to be included in this review and not to include other studies?
  *Response:* For Table 2, the limitations column was removed and replaced with a new column describing how medication adherence was being monitored in each study. These studies were often very fundamental, so often they are being conducted to determine if the technology can differentiate between medication-taking actions or other unrelated behaviors. This new column is titled data analyzed using machine learning to determine adherence. For [5], they are using movement sensors in addition to the traditional cap sensor used in many adherence studies in the past. This is a potential stepping-stone toward monitoring medication adherence with devices that people wear in their everyday lives like a smartwatch. The movement sensors are providing movement data similar to a smartwatch but also provide cap sensor data, which is more reliable at this time for determining medication ingestion. This information can be used together to determine when the movement data alone is actually able to accurately report medication ingestion.
- Table 3: Same as above, plus which aspect of adherence was improved—the one that was tested; the other one?
  *Response:* I agree that the title of the table is confusing and have changed it to summary of studies that monitor and attempt to improve medication adherence. These studies all introduce some type of intervention to hopefully improve medication adherence but did not necessarily accomplish their goal. Like Table 2, I have removed the limitations column and replaced it with a column titled data analyzed using machine learning to determine adherence. I believe that this new column provides a lot of useful information that makes understanding the purpose of each study much easier.
- Line 210, 213: Correct “99 DOTS” to “99DOTS.”
  *Response:* I have corrected to 99DOTS.
- Line 221, 222: “The next paper used face recognition software and computer vision to monitor medication adherence”—which aspect of medication adherence are you considering here?
  *Response:* I have added more detail to this part of the paper to make it more clear. This study used machine learning to monitor medication adherence for clinical trials and to predict which patients were not likely to be adherent over the duration of the study. This system also provided reminders to patients to help maintain or improve their adherence.
- Line 241-2: “These assessments were then used to create predictors”—predictors of what? I guess not of medication adherence, if you say that medication adherence was a...predictor!
  *Response:* One of the strongest predictors of future medication adherence in this study was medication adherence of the same patient in the past. I have added the words past and future to make this distinction more clear.
- Line 247-50: Usually, limitations are provided at the end of the Discussion.
  *Response:* I agree with this point and have moved the Limitations after the Discussion and Conclusions.
- Line 285-6 states: “Approximately 87% of these studies used either logistic regression, artificial neural networks, support vector machines, or random forest algorithms.” Why is this not visible in Table 1?
  *Response:* This is portrayed in Table 1 but not in the form of a summarized percentage. If you add up the number of studies that are using at least one of these algorithms and then divide this by the total number of studies in this group, you get 87%. It would be difficult to put this on the table in a way that is easily understandable, which is why I stated it in the text.
- Lines 282 and 342 still use the term “compliance” instead of “adherence.”
  *Response:* I have corrected these terms from compliance to adherence.
- Lines 288-291: You provide comparisons of the accuracy of diverse algorithms yet without any statistical significance values. That sort of simple comparison is not inconclusive.
  *Response:* In the Discussion and Conclusions section, I acknowledge that no meaningful conclusions can be drawn from these comparisons. I only included this part in the paper because someone that I presented my work to previously asked if any of the studies compared the accuracy of different algorithms. Some of the studies did do this, so I thought I should briefly mention it to show that not much work has been accomplished yet on this front.

### Reviewer BQ

I am appreciative that the author was willing to do the changes, and the manuscript has improved.

Although this is a scoping review, I would like to find out how the author ensured robustness and reproducibility. As of now, with the study design largely using one author, there is no way to assess if the paper selection is robust or independent. I strongly feel that there is a need for 2 authors to independently select studies, even though it is a scoping review, to give this review some robustness. If not, how different will the results be compared to a narrative review?

*Response:* This study was created using the insight of multiple people and I have added a lot of detail to better illustrate their role in creating this work throughout the Methods section. Additionally, I have added another coauthor that independently repeated the title/abstract and full-text reviews. They also evaluated the grouping of studies to increase the robustness of the review.

## Abbreviations

IT: information technology
PRISMA: Preferred Reporting Items for Systematic Reviews and Meta-Analyses

## References

[1] Kardas P (2021). Peer review of "Machine Learning and Medication Adherence: Scoping Review". JMIRx Med.

[2] Bohlmann A, Mostafa J, Kumar M (2021). Machine learning and medication adherence: scoping review. JMIRx Med.

[3] Vrijens B, De Geest S, Hughes DA, Przemyslaw K, Demonceau J, Ruppar T, Dobbels F, Fargher E, Morrison V, Lewek P, Matyjaszczyk M, Mshelia C, Clyne W, Aronson JK, Urquhart J, ABC Project Team (2012). A new taxonomy for describing and defining adherence to medications. Br J Clin Pharmacol.

[4] Kwan YH (2021). Peer review of "Machine Learning and Medication Adherence: Scoping Review". JMIRx Med.

[5] Aldeer M, Alaziz M, Ortiz J, Howard RE, Martin RP (2019). A sensing-based framework for medication compliance monitoring. Proceedings of the 1st ACM International Workshop on Device-Free Human Sensing.

